# Radiomic Machine Learning and External Validation Based on 3.0 T mpMRI for Prediction of Intraductal Carcinoma of Prostate With Different Proportion

**DOI:** 10.3389/fonc.2022.934291

**Published:** 2022-06-28

**Authors:** Ling Yang, Zhengyan Li, Xu Liang, Jingxu Xu, Yusen Cai, Chencui Huang, Mengni Zhang, Jin Yao, Bin Song

**Affiliations:** ^1^ Department of Radiology, West China Hospital of Sichuan University, Chengdu, China; ^2^ Department of Radiology, Sichuan Cancer Hospital and Institute, Sichuan Cancer Center, School of Medicine, University of Electronic Science and Technology of China, Chengdu, China; ^3^ Department of Research Collaboration, R&D center, Beijing Deepwise & League of PHD Technology Co., Ltd., Beijing, China; ^4^ Department of Pathology, West China Hospital of Sichuan University, Chengdu, China

**Keywords:** radiomics, machine learning, prostate cancer, intraductal carcinoma, multiparametric MRI

## Abstract

**Purpose:**

To assess the association of radiomics features based on multiparametric MRI (mpMRI) with the proportion of intraductal carcinoma of prostate (IDC-P) and validate the predictive models.

**Materials and Methods:**

We retrospectively included pre-treatment MR images of prostate cancer (PCa) with IDC components of high proportion (≥10%, hpIDC-P), low proportion (<10%, lpIDC-P), and pure acinar adenocarcinoma (PAC) from our institution for training and internal validation and cooperated cohort for external validation. Normalized images of T2WI, diffusion weighted imaging (DWI) and apparent diffusion coefficient (ADC) map, and dynamic contrast enhanced (DCE) sequences were used for radiomics modeling. The clinical model was built based on serum total prostate specific antigen (tPSA) and Gleason score (GS), and the integrated model was the combination of Rad-score and clinicopathological data. The discrimination ability was assessed by area under the receiver operating characteristic curve (ROC-AUC) in the internal and external validation sets and compared by DeLong test.

**Results:**

Overall, 97 patients with hpIDC-P, 87 lpIDC-P, and 78 PAC were included for training and internal validation, and 11, 16, and 19 patients for external validation. The integrated model for predicting hpIDC-P got the best ROC-AUC of 0.88 (95%CI = 0.83-0.93) in internal and 0.86 (95%CI = 0.72-1.0) in external validation, which both outperformed clinical models (AUC=0.78, 95% CI = 0.72-0.85, AUC=0.69, 95% CI = 0.5-0.85, respectively) based solely on GS, and the radiomics model (AUC=0.85, 95% CI = 0.79-0.91) was slightly inferior to the integrated model and better than the clinical model in internal dataset. The integrated model for predicting lpIDC-P outperformed both radiomics and clinical models in the internal dataset, while slightly inferior to the integrated model for predicting hpIDC-P.

**Conclusions:**

Radiomics signature improved differentiation of both hpIDC-P and lpIDC-P versus PAC when compared with the clinical model based on Gleason score, and was validated in an external cohort.

## Introduction

Globally, prostate cancer (PCa) is the most commonly diagnosed cancer in men and ranks among the top five cancers for both incidence and mortality (1). Interestingly, the 5-year survival rate is *>* 60% in localized PCa, while the remaining ≈ 5% in patients with distant metastasis (2). Intraductal carcinoma of the prostate (IDC-P), which was recently defined by the World Health Organization (WHO) in 2016, is a distinct clinicopathological entity characterized by malignant prostatic epithelial cells growing within preexisting ducts and/or acini. In addition, it is associated with aggressive biological behavior and a clinical course (3, 4). Most IDC-P tumors are considered to represent the intraductal spread of adenocarcinoma and are almost always adjacent to invasive acinar adenocarcinoma rather than occurring on their own. Therefore, it is difficult to achieve an accurate diagnosis *via* random sampling and categorize its growth patterns (4, 5). Although previous studies have confirmed that multiparametric MRI (mpMRI)/ultrasound fusion targeted biopsy can enhance the detection of IDC-P (6), as well as the fact that Gleason score (GS) 7 PCa with IDC showed lower apparent diffusion coefficient (ADC) values than those without IDC by using a small sample size (15 vs. 15 cases) (7), the difference of overall intra-tumoral signal characteristics between IDC-P and pure prostatic acinar adenocarcinoma (PAC) have not yet been reported. Moreover, Zhao et al. reported that a 10% or greater proportion of IDC of the prostate is an unfavorable prognosticator for metastatic PCa (8).

Radiomics is the high-throughput extraction and mining quantitative textures and features (region size, shape or location, histogram of volume intensity, texture analysis, transform analysis, fractal analysis) from a large amount of medical imaging through semi-automatic or automatic software (9). It offers the potential to analyze whole tumors in 3D, as well as sub-regions or ‘habitats’ within tumors. More recently, the integration of radiomics and genomics has developed radiogenomics, which specifically investigates the associations between quantitative imaging features and characteristics typically attributed to the genomics domain and its immediate derivatives (9) Artificial intelligence is the field of computer science aimed at making smart devices that perform tasks that currently require human intelligence (10). Machine learning is a subfield of artificial intelligence and is based on the development and training of algorithms, by which computers may learn from data and perform predictions without specific prior programming (10, 11). Unlike classical rule-based algorithms, it can take advantage of increased exposure to large and new data as well as to improve and learn over time (11). With the utilization of artificial intelligence and machine learning, it expanded the directions of prostatic radiomics and mainly focused on gland segmentation, assessing lesion aggressiveness, identification of tumors through mpMRI, creation of frameworks for automated PCa localization and detection, prediction of prognosis and progression, and finally management of treatment and toxicity (9, 10).

Thus, the purpose of this study was to differentiate high proportion and low proportion IDC-P (hpIDC-P and lpIDC-P, respectively) from PAC by using radiomic machine learning of mpMRI. Further, an external cohort of an independent dataset was used to validate the constructed models.

## Materials and Methods

### Study Population

**Figure 1:**
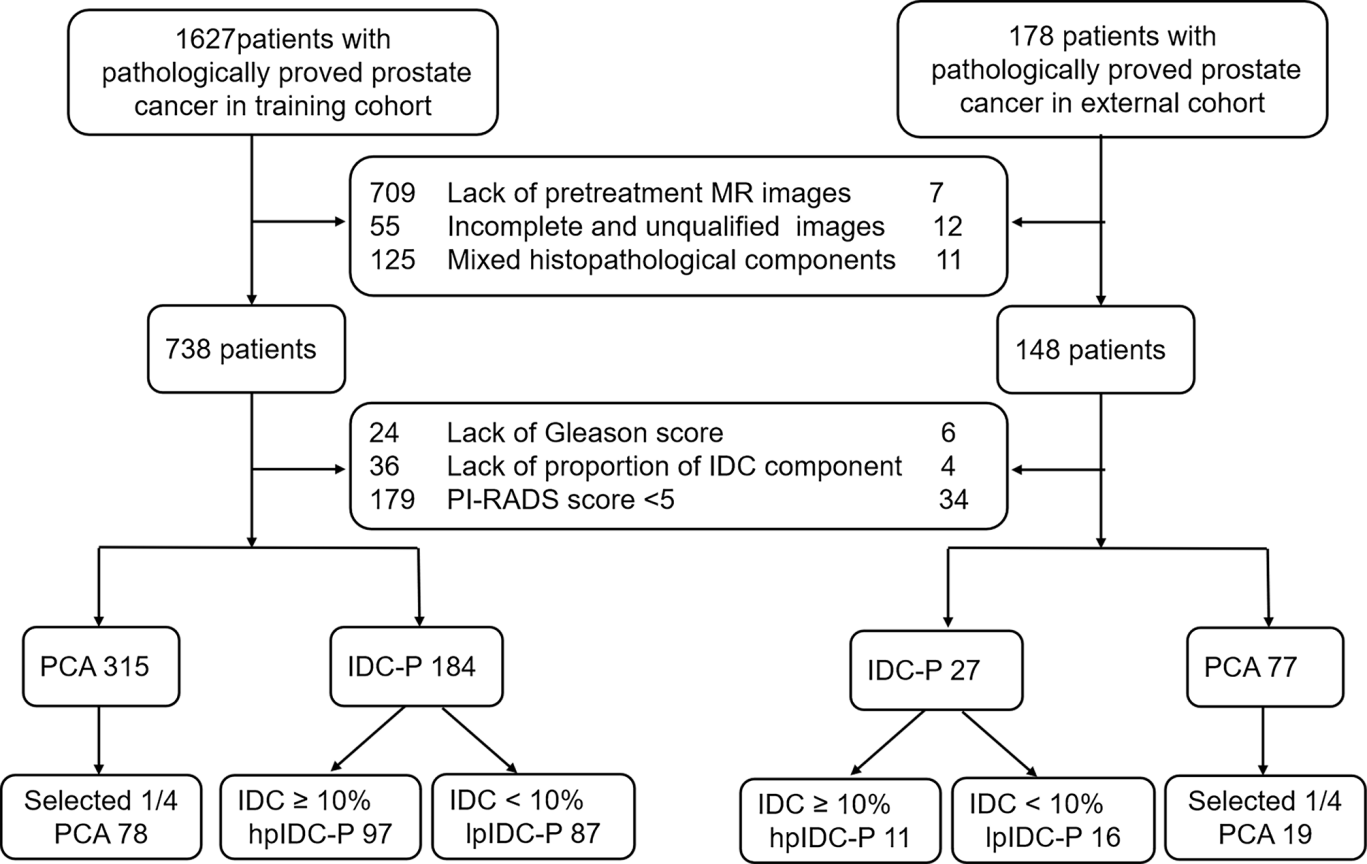
Diagram for the patient selection process. IDC, intraductal carcinoma; PAC, prostatic acinar adenocarcinoma; PI-RADS, Prostate Imaging Reporting and Data System.

This retrospective multicenter study was approved by the local Institutional Review Boards (No. 2019-1209), and written informed consent was waived. Consecutive patients with pathologically proven IDC-P with a proportion of at least 12 core needle biopsies or radical specimens were enrolled from January 2015 to December 2020 for the analysis. The proportion of IDC was calculated as the percent of the total area of IDC in the total area of the entire PCa, which was primarily based on radical specimens without utilizing neoadjuvant hormonal therapy (NHT) and followed by puncture specimens. The PAC group included simultaneous diagnoses *via* both needle biopsy without NHT and radical specimen with or without NHT, and one-fourth of the patients were randomly selected at our institution for modeling. All biopsies were performed with systematic or cognitive mpMRI target biopsy with at least 12 punctures. The external validation set was collected from May 2019 to December 2020 from two cooperating institutions. All the included mpMRI were comprised of pretreatments and with lesions possessing a Prostate Imaging-Reporting and Data System (PI-RADS) score of 5 according to the standard of 2019 version 2.1. The exclusion criteria included prior local or systemic therapies for any prostate abnormality before the examination, any other mixed histopathological components (ductal adenocarcinoma, urothelial carcinoma, squamous cell carcinoma, neuroendocrine carcinoma, mucinous adenocarcinoma, or cacinosarcoma) or patients performed on 1.5T MRI scanners, as well as incomplete sequences or poor quality of mpMRI. The process of patient enrollment is shown in **Figure 1**.

### MRI Acquisition

All the MR images from the training cohort were obtained by 3.0 T MRI scanners obtained from two vendors (Vendor 1: Skyra 3.0 T from Siemens, Erlangen, Germany; Vendor 2: DISCOVERY MR750w 3.0 T from GE Healthcare, Chicago, IL, USA), and the external cohort images were obtained by two venders (Vendor 1: Skyra 3.0 T from Siemens, Erlangen, Germany; Vendor 3: uMR780 from United Imaging Health care, Shanghai, China) with a pelvic phased array coil. The imaging parameters were performed as follows: axial and sagittal T2-weighted turbo spin-echo (TSE) sequence with small field of view (FOV) of 240 mm, repetition time (TR)/echo time (TE) of 3235 - 6220 ms/104.0 - 149.4 ms, flip angle of 103.5 - 160°, slice thickness of 3.0 - 3.5 mm and bandwidth of 122 - 260 Hz/Px; transverse diffusion-weighted imaging (DWI) sequence of single-shot echo planar imaging with FOV of 240-320 mm, TR/TE of 2083 - 5,100 ms/81.5 - 89 ms, slice thickness of 3.0 - 3.5 mm, bandwidth of 1202 - 1953 Hz/Px and b values of 50, 200, 800, and 1400 s/mm^2^, the ADC map was automatically calculated; transverse 3D-fast field echo of dynamic contrast-enhanced (DCE) sequence with a temporal resolution of 15 s after intravenous injection of 0.1 mmol/kg gadolinium chelate at a rate of 2 mL/s, FOV of 240 - 380 mm, TR/TE of 3.92 - 4.08 ms/1.69 - 1.90 ms, flip angle, 9° - 12°, slice thickness, 2 - 3 mm, bandwidth, 440 - 600 Hz/Px, measurement, 7.

### Clinicopathological Data Collection

The baseline clinicopathological characteristics, including age, biopsy according to the International Society of Urological Pathology (ISUP) grade group (GG), and serum total prostate specific antigen (tPSA), of both the training and external validation cohorts, were collected. The ISUP GG of the five-tier grading system was used to record the Gleason score as follows: GG 1 for GS 6, GG 2 for 3 + 4, GG 3 for 4 + 3, GG 4 for 8, and GG 5 for 9 or 10 (12). Pretreatment tPSA was categorized as the following groups because there was no exact value when it exceeded 100 ng/mL in our institution before 2018: < 10 ng/mL, 10 - 19.9 ng/mL, 20 - 99.9 ng/mL and ≥ 100 ng/mL (13). Clinical models for predicting hpIDC-P and lpIDC-P were built and externally validated.

### Feature Extraction and Selection

All the mpMRIs, including axial T2WI, DWI with a high b value of 1400 s/mm^2^ and ADC map, DCE image with the most obvious enhanced tumor, were reviewed by two experienced radiologists with 9 years of experience (reader 1, L.Y.) and 6 years of experience (reader 2, Z.Y.L.) in making PCa diagnoses to ensure the existence of one index lesion with PI-RADS 5 for each patient. The lesions were then semi-automatically segmented for identifying the boundary of the tumors by reader 1 who was blinded to the clinicopathological information. Any disagreement was resolved *via* discussion or consultation with another senior radiologist with 16 years’ experience (reader 3, J.Y.) in making genitourinary system diagnoses.

The images were normalized automatically on the Research Platform (Deepwise Inc., Beijing, China, http://keyan.deepwise.com) before feature calculation. Radiomic features of the lesions were extracted using the Python package PyRadiomics (version 2.2.0). In detail, each image was subtracted by the mean value and was divided by the standard deviation value. Afterward, the image was multiplied by 100 and resampled to the same resolution. The volume of interest (VOI) of each lesion was automatically extracted. A total of 2107 features (**Supplementary Table S1**), including 414 first-order features, 14 shape features, and 1679 texture features, were extracted from the VOI of each sequence. *via* Fifteen patients from each group (for a total of 45 patients in the training institution) were randomly resampled for the interclass (readers 1 and 2) and intraclass (reader 1 twice at an interval of 1 month) correlation coefficient (ICC) analyses. The radiomic features were retained after ICC analysis and Pearson correlation coefficients analysis. Subsequently, the least absolute shrinkage and selection operator (LASSO) regression method was applied to select the most distinguishable features. During the feature selection process, non-zero coefficient after the shrinking process was selected.

### Radiomics and Integrated Model Construction

After feature selection, the support vector machine (SVM) regression was used to construct the radiomics model. A five-fold internal cross-validation was performed on the training set to determine the optimal parameter configuration for the radiomics model, after which it was validated by the external dataset. After the radiomic model was built for predicting hpIDC-P and lpIDC-P, the corresponding Rad-score in the training and internal validation datasets were calculated and combined with the variables of the clinical model for training and validating the integrated model by using logistic regression. Each clinical feature was assessed by univariate logistic regression. The features revealed as statistically significant with univariate logistic regression analysis were then analyzed with multivariate logistic regression analysis for integrated model-building.

**Figure 2:**
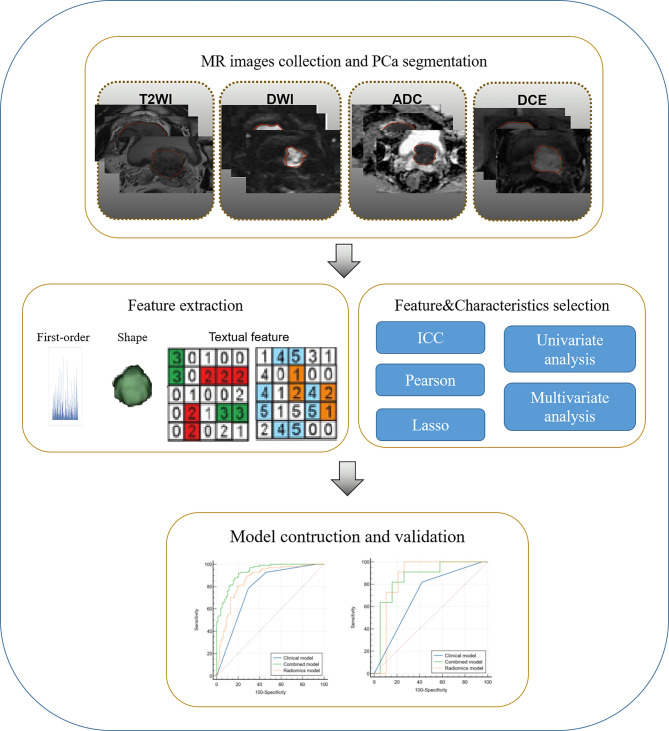
Radiomics workflow.

Additionally, an external dataset was used for validation. In addition, both the performance of the internal and external validation of the clinical, radiomics and integrated models were compared in the hpIDC-P and lpIDC-P predictive models, respectively, as shown in **Figure 2**.

### Statistical Analysis

All the statistical analyses were performed using R software (version: 3.6.1) and MedCalc Version 20.0.22 (MedCalc, Ostend, Belgium). Differences in clinicopathological parameters were assessed by the chi-squared test for categorical variables and the Mann–Whitney U test for continuous variables. The radiomic features with ICC > 0.75 were retained for the analysis. Redundant features were removed by Pearson correlation coefficients with a threshold value of 0.8. For the establishment of clinical model, univariate logistic regression was used to initially select the clinical predictors, after which those variables with *P* < 0.05 were introduced into a multivariate logistic regression. The receiver operating characteristic (ROC) curves of different models were compared by the DeLong test. Statistical significance was set at *P* < 0.05.

## Results

### Patient Characteristics

Overall, 262 patients from our institution, including 97 hpIDC-P, 87 lpIDC-P and 78 PAC patients, met all the criteria and were used for training and internal validation. Forty-six patients from cooperating institutions were assigned for external validation, including 11 hpIDC-P, 16 lpIDC-P and 19 PAC cases.

The clinicopathological characteristics and statistical results are shown in **Table 1**. There were no significant differences in patient age or tPSA distribution (all *P* > 0.05) among any of the subgroups in the training and external validation cohorts. There was a significant difference in biopsy GG in the training cohort (*P* < 0.001), whereas there was no significant difference in the external validation cohort (*P >* 0.05).

**Table 1:** The clinicopathological data of patients in training and external validation cohorts.

	Training cohort			External validation cohort		
Group	PCA(78)	lpIDC-P(87)	hpIDC-P(97)	PCA(19)	lpIDC-P(16)	hpIDC-P(11)
Age,y (IQR)	69 (64-74)	69 (63-73)	68 (62-75)	74 (71-78)	69 (65-76)	72 (66-79)
tPSA,ng/mL						
<10	8 (10.3)	4 (4.6)	10 (10.3)	0 (0.0)	0 (0.0)	0 (0.0)
≥10 <20	21 26.9)	14 (16.1)	15 (15.5)	2 (10.5)	0 (0.0)	0 (0.0)
≥20 <100	34 (43.6)	46 (52.9)	39 (40.2)	10 (52.6)	7 (43.7)	3 (27.3)
≥100	15 (19.2)	23 (26.4)	33 (34.0)	7 (36.8)	9 (56.3)	8 (72.7)
biopsy GG		*	*			
2 (GS3+4)	6 (7.7)	2 (2.3)	0 (0.0)	1 (5.3)	0 (0.0)	0 (0.0)
3 (GS4+3)	36 (46.2)	11 (12.6)	7 (7.2)	5 (26.3)	0 (0.0)	1 (9.1)
4 (GS8)	13 (16.7)	11 (12.6)	14 (14.4)	5 (26.3)	5 (31.3)	1 (9.1)
5 (GS≥9)	23 (29.5)	63 (72.4)	76 (78.4)	8 (42.1)	11 (68.7)	9 (81.8)

*P < 0.001 lpIDC-P vs. PAC and hpIDC-P vs. PAC in training cohort.

### Clinical Model

Among the clinicopathological parameters, only biopsy GG was a predictor of hpIDC-P (OR = 4.107, 95% CI, 2.67-6.319, *P* < 0.001) and lpIDC-P (OR = 2.714, 95% CI, 1.877-3.924, *P* < 0.001) in the training set *via* multivariate logistic regression and was used to build the clinical model. The AUCs of the clinical model were 0.78 (95% CI, 0.72-0.85) for predicting hpIDC-P and 0.74 (95% CI, 0.66-0.81) for lpIDC-P in the training set; in addition, the AUCs were 0.69 (95% CI, 0.5-0.85) and 0.68 (95% CI, 0.50-0.83) in the external validation set, respectively.

### Radiomics Model

After excluding features with ICC values ≤ 0.75 including 606 features of T2WI, 681 features of DWI, 362 features of ADC, and 382 features of DCE, 5589 features of the above mentioned four sequences were retained and used for the follow-up analysis.

For the radiomics model of hpIDC-P and PAC differentiation, there were four features in DCE, two features in T2WI, four features in DWI, and two features in ADC, for a total of 12 features (**Supplementary Table S2**), which contained six gray level cooccurrence matrix (GLCM), four gray level dependence matrix (GLDM), and two gray level run length matrix (GLRLM) features after using the LASSO regression. The AUCs of the training set, internal validation set, and external validation set were 0.95 (95% CI, 0.92-0.98), 0.85 (95% CI, 0.79-0.91), and 0.86 (95% CI, 0.72-1.0), respectively.

For the radiomics model of lpIDC-P and PAC differentiation, there were seven features in DCE, five features in T2WI, and three features in DWI, for a total of 15 features (**Supplementary Table S3**), which contained nine GLCM, three GLDM, two GLRLM, and one gray level size zone matrix (GLSZM) features after using the LASSO regression. The AUCs of the training set, internal validation set, and external validation set were 0.86 (95% CI, 0.81-0.92), 0.8 (95% CI, 0.73-0.87), and 0.74 (95% CI, 0.56-0.91), respectively.

### Clinical-Radiomics Integrated Model

The clinical-radiomics integrated model incorporating biopsy GG and Rad-score obtained AUCs of 0.88 (95% CI, 0.83-0.93) in the internal validation set and 0.86 (95% CI, 0.72-1.0) in the external validation set of the hpIDC-P model; in addition, there were AUCs of 0.83 (95% CI, 0.76-0.89) and 0.76 (95% CI, 0.59-0.92) for the lpIDC-P model, respectively.

### Comparison of Performance Between Different Models

**Figure 3:**
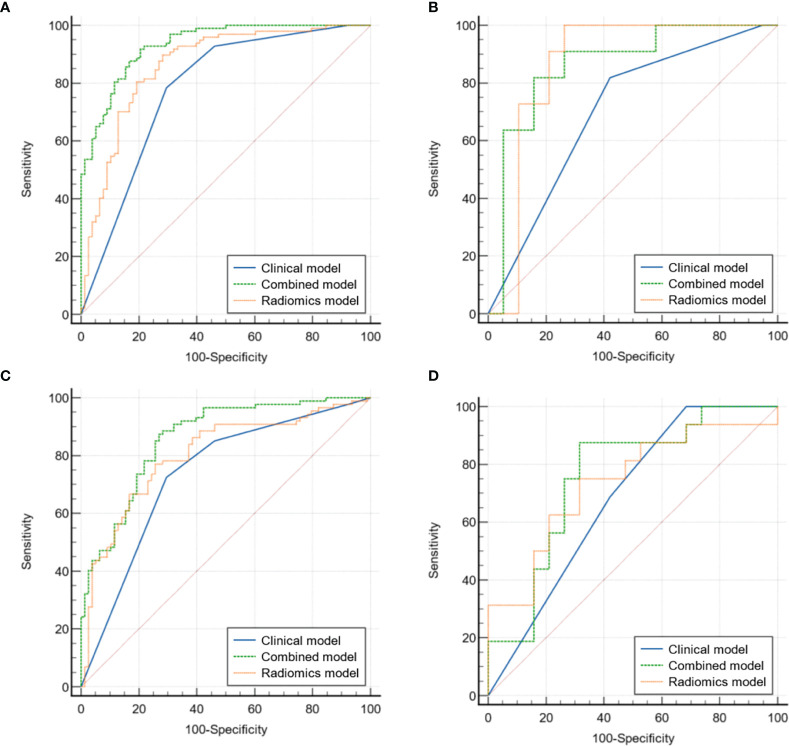
Comparison of ROC curves for the validation of clinical, radiomics, and integrated models for predicting hpIDC-P (**A** internal, **B** external) and lpIDC-P (**C** internal, **D** external).

**Table 2** and **Figure 3** detail the comparison results on the AUC and ROC curves of different models in the internal and external validation sets. For predicting hpIDC-P, both radiomics and integrated models had significant advantages compared to the clinical model, and the integrated model outperformed the radiomics model, with statistical significance being observed in the internal validation set (all *P* < 0.05). The integrated model also outperformed the clinical model with statistical significance (*P* < 0.05), whereas there was no significant difference between the radiomics model and integrated and clinical models in the external validation set (both *P* > 0.05). For predicting lpIDC-P, integrated models had significant advantages compared to both radiomics and clinical models (both *P* < 0.05), whereas the radiomics model was not significantly different from the clinical model (*P* > 0.05) in the external validation set. There were no significant differences between any two models.

**Table 2:** The performance of internal and external validation of predicting hpIDC-P and lpIDC-P.

Model	Internal validation			External validation		
hpIDC-P vs. pPAC	AUC	95% CI	Youden index	AUC	95% CI	Youden index
Clinical Model	0.78	0.72-0.85	0.489	0.69	0.5-0.85	0.359
Radiomics Model	0.85*	0.79-0.91	0.564	0.86	0.72-1.0	0.646
Integrated Model	0.88*^#§^	0.83-0.93	0.587	0.86*	0.72-1.0	0.737
lpIDC-P vs. pPAC	AUC	95% CI	Youden index	AUC	95% CI	Youden index
Clinical Model	0.74	0.66-0.81	0.429	0.68	0.50-0.83	0.266
Radiomics Model	0.8	0.73-0.87	0.474	0.74	0.56-0.91	0.382
Integrated Model	0.83*^#^	0.76-0.89	0.501	0.76	0.59-0.92	0.401

*P < 0.05 vs Clinical model, ^#^P < 0.05 vs Radiomics model. ^§^P = 0.030 vs. lpIDC-P model.

The verification of the hpIDC-P outperformed the lpIDC-P models between the same type of model without a significant difference (all *P* > 0.05), except for the integrated model of the internal validation set (*P* = 0.030).

Overall, both radiomics and integrated models achieved good performance for predicting hpIDC-P and lpIDC-P in the internal and external validation sets, and the effectiveness of the integrated model of hpIDC-P was even better than that of lpIDC-P in the internal validation set.

## Discussion

IDC-P is an aggressive type of prostate cancer, which is independently associated with the adverse clinical and pathological features on both biopsy and radical prostatectomy. In these patients, there are higher Gleason score and likelihood of seminal vesicle invasion, as well as worse progression-free, cancer-specific, and overall survival (14). Furthermore, patients with IDC-P on radical prostatectomy have higher pathologic T stage, increased likelihood of extraprostatic extension, and increased risk of biochemical recurrence and metastasis (14). Therefore, diagnosis and early reporting is critical for choosing the most appropriate patient management. The majority of IDC-P lesions have been identified in close proximity to invasive carcinoma of Gleason pattern 4 or 5, rather than in formation of a single solid tumor (4), which has made it difficult to be macroscopically diagnosed by radiologists. The advantages of radiomic machine learning techniques *via* detailed analyses of tumor morphology and internal texture features may be helpful to assist radiologists in the diagnosis of IDC-P. In this retrospective study, we built clinical, radiomics, and clinical-radiomics integrated models to identify IDC-P with high and low proportions and compared the verification effectiveness among these models. Overall, the integrated model showed good predictive performance *via* internal cross and external validation for predicting both hpIDC-P and lpIDC-P, especially for hpIDC-P.

All the final features used for radiomics modeling in this study were based on the semi-automatic sketching mode and PI-RADS 5 score lesions which could help ensure excellent inter-reader agreement (15), and were gray level matrix features for predicting hpIDC-P and lpIDC-P. First, IDC components were scattered in the entire tumor, which resulted in a certain correlation and regularity in the distribution of signal intensity among the voxels. Second, the included cases of PCa in this study mainly consisted of Gleason patterns 4 and 5. The difference in first-order statistical features based on the intensity distribution of individual voxels may not be so obvious between the groups. Third, PCa has a relatively unique nondestructive infiltration pattern of intervening stroma between benign preexisting glands, thus creating irregular tongues of tumor tissue that form scattered and irregular shapes regardless of GS (16). Therefore, the value of the morphological features in distinguishing subgroups of PCa is limited.

In previous studies, radiomics models based on DWI sequences were more efficient in predicting high-grade PCa, whereas the T2WI sequence was more likely to differentiate low-grade PCa with similar grades (17–21). The possible reason for this phenomenon is that the DWI sequence or ADC map may be more sensitive to detect the changes in restricted diffusion of water molecules caused by high-grade PCa, whereas T2WI was better at demonstrating the signal changes caused by slight differences in PCa grade through its high tissue resolution, which was consistent with our findings. The radiomic features of the hpIDC-P model were more based on DWI and ADC maps, whereas the lpIDC-P model was greater on T2WI. In addition, both radiomics models were highly counted on the DCE sequence. Bleker et al. found that when removing DCE features from the combined T2WI+DWI+DCE model to quantify clinically significant PCa, the AUC would be reduced from 0.870 to 0.816 (22). Although there is a current trend of highlighting the usefulness of biparametric MRI by omitting DCE imaging from routine mpMRI to diagnose PCa (23, 24), our study suggested that DCE features may play a role in identifying subtypes of PCa.

Several studies on predictive models of high-grade (GS > 7) PCa, clinically significant PCa, and extraprostatic extension have shown similar results, in that the radiomics model outperformed the clinical model and was comparable with the clinical-radiomics combined model (18, 25, 26). The internal validation efficiency of the integrated models of both hpIDC-P and lpIDC-P were significantly outperformed the clinical and radiomics models, as did the radiomics model of hpIDC-P outperformed the clinical model, but not the model of lpIDC-P. Moreover, our results showed that the addition of radiomics signature with the clinical model improved the differentiation of both hpIDC-P and lpIDC-P versus PAC. Conversely, themportantce of ISUP GG for the prediction of IDC-P (especially for lpIDC-P) was based on the fact that GS would increase when the occurrence of IDC encountered invasive carcinoma, according to the 2016 ISUP consensus. From the perspective of microscopic structure of tumor cells, most lpIDC-P in our study had 2% or 5% of IDC components scattered in the entire tumor, which was closer to the PAC than hpIDC-P. Therefore, the difficulty was increased for identifications *via* the sole use of radiomic matrix features. In the future, the integration of patient socio-demographic, clinical, and biochemical characteristics in the pathway of the imaging-based radiomics features might help to increase their clinical practicability (27).

There are some limitations in this study. First, the main limitation of this study was the retrospective nature of failing to unify the scanning protocols among MRI acquisition which could represent a potential bias, as well as the fact that the proportion of IDC-P was solely quantified by using biopsy specimens, as some patients have no opportunity for radical prostatectomy. To improve the quality of the tissue sampled for pathological evaluation, several targeted biopsy (TBx) techniques are currently available, including MRI-TRUS (transrectal ultrasound) fusion TBx, trans-perineal fusion biopsy, MRI In-bore TBx (both trans-rectal and trans-perineal), and cognitive registration TRUS-TBx (28). Second, another limitation was that the small study population of the external cohort, which was mainly caused by the importance of IDC-P diagnoses, has not yet been fully realized. Third, the inclusion of the PAC group was randomly selected rather than using consecutive patients because of the considerable difference in the sample sizes of IDC-P and PAC. Finally, we excluded cases with a PI-RADS score less than 5 mainly because small or uncertain tumor foci would add a more unexplainable radiomics signature for this issue.

In conclusion, the radiomics signature improved the differentiation of both hpIDC-P and lpIDC-P versus PAC compared with the clinical model based on the Gleason score. The integrated model for predicting hpIDC-P also outperformed the clinical model in the external validation set.

## Data Availability Statement

The raw data supporting the conclusions of this article will be made available by the authors, without undue reservation.

## Ethics Statement

The studies involving human participants were reviewed and approved by Biomedical Research Ethics Committee of West China Hospital. The ethics committee waived the requirement of written informed consent for participation.

## Author Contributions

LY and ZL: Data curation, software, formal analysis, validation, investigation, methodology, writing original draft. XL: Resources, data curation, software, formal analysis, validation, investigation, methodology. JX, YC, and CH: Software, formal analysis, validation, investigation, methodology, visualization. MZ: Conceptualization, data curation, formal analysis, validation, methodology. JY and BS: Conceptualization, resources, data curation, formal analysis, supervision, validation, investigation, methodology, project administration, writing review and editing. All authors contributed to the article and approved the submitted version.

## Funding

This work was supported by the 1*3*5 Project for Disciplines of Excellence, West China Hospital, Sichuan University (No. ZY2017304), Science and Technology Support Program of Sichuan Province (No. 2022NSFSC0840) and the sky imaging research foundation (Z-2014-07-1912).

## Conflict of Interest

Authors JX, YC, and CH are employed by Beijing Deepwise & League of PHD Technology Co., Ltd.

The remaining authors declare that the research was conducted in the absence of any financial relationships that could be construed as a potential conflict of interest

## Publisher’s Note

All claims expressed in this article are solely those of the authors and do not necessarily represent those of their affiliated organizations, or those of the publisher, the editors and the reviewers. Any product that may be evaluated in this article, or claim that may be made by its manufacturer, is not guaranteed or endorsed by the publisher.
