# Corrigendum: Radiomic machine learning and external validation based on 3.0T mpMRI for prediction of intraductal carcinoma of prostate with different proportion

**DOI:** 10.3389/fonc.2024.1401121

**Published:** 2024-04-03

**Authors:** Ling Yang, Zhengyan Li, Xu Liang, Jingxu Xu, Yusen Cai, Chencui Huang, Mengni Zhang, Jin Yao, Bin Song

**Affiliations:** ^1^Department of Radiology, West China Hospital of Sichuan University, Chengdu, China; ^2^Department of Radiology, Sichuan Cancer Hospital and Institute, Sichuan Cancer Center, School of Medicine, University of Electronic Science and Technology of China, Chengdu, China; ^3^Department of Research Collaboration, R&D center, Beijing Deepwise & League of PHD Technology Co., Ltd., Beijing, China; ^4^Department of Pathology, West China Hospital of Sichuan University, Chengdu, China

**Keywords:** radiomics, machine learning, prostate cancer, intraductal carcinoma, multiparametric MRI

In the published article, there was an error in the Funding statement for Science and Technology Support Program of Sichuan Province (No. 22NSFSC117). The correct Funding statement appears below.

**FUNDING**

This work was supported by the 1*3*5 Project for Disciplines of Excellence, West China Hospital, Sichuan University (No. ZY2017304), Science and Technology Support Program of Sichuan Province (No. 2022NSFSC0840) and the sky imaging research foundation (Z-2014-07-1912).

The authors apologize for this error and state that this does not change the scientific conclusions of the article in any way. The original article has been updated.

